# Metallic ground states of undoped Ti_2_O_3_ films induced by elongated *c*-axis lattice constant

**DOI:** 10.1038/s41598-020-79182-5

**Published:** 2020-12-17

**Authors:** K. Yoshimatsu, N. Hasegawa, Y. Nambu, Y. Ishii, Y. Wakabayashi, H. Kumigashira

**Affiliations:** 1 Institute of Multidisciplinary Research for Advanced Materials, Tohoku University, Sendai, Miyagi 980-8577 Japan; 2 Materials Research Center for Element Strategy (MCES), Tokyo Institute of Technology, Yokohama, 226-8503 Japan; 3 Institute for Materials Research, Tohoku University, Sendai, Miyagi 980-8577 Japan; 4 Department of Physics, Tohoku University, Sendai, Miyagi 980-8578 Japan; 5 Photon Factory, Institute of Materials Structure Science, High Energy Accelerator Research Organization (KEK), 1-1 Oho, Tsukuba, 305-0801 Japan

**Keywords:** Electronic properties and materials, Phase transitions and critical phenomena, Surfaces, interfaces and thin films

## Abstract

Ti_2_O_3_ exhibits unique metal–insulator transition (MIT) at ~ 450 K over a wide temperature range of ~ 150 K. The close relationship between MIT and crystal deformation has been proposed. However, as physical properties are governed by the thermodynamic equilibrium in bulk systems, conducting experimental studies under different lattice deformations remains challenging. Epitaxial thin films can offer high flexibility to accommodate adaptive crystal lattices and provide efficient platforms for investigating the MIT. In this study, we report the synthesis of corundum-type Ti_2_O_3_ films on various growth temperatures. We found that the metallic ground states appeared in the films grown at low temperatures. The electronic ground states were further investigated by the electronic-structure calculations. Results suggest that the electrical properties of Ti_2_O_3_ films were governed by the *c*/*a* ratio of the crystal structure, and the absence of the MIT was attributed to the lattice deformation characterized by an elongated *c* lattice constant.

## Introduction

Ti_2_O_3_ with a corundum-type crystal structure exhibits unique metal–insulator transition (MIT). It is a nonmagnetic insulator with a small bandgap energy of ~ 100 meV^1^ at low temperatures, and exhibits transition to metallic states at high temperatures, i.e., ~ 450 K^2–9^, which extends over a broad temperature of ~ 150 K. This is indeed unique than most other transition-metal oxide systems. Although the crystal symmetry remains unchanged across the MIT, the *c*/*a* ratio of unit cell changes significantly, thereby suggesting a close relationship between the MIT and lattice deformations^4–9^.

The MIT mechanism has been discussed based on experimental and theoretical investigations^1–22^. The most relevant phenomenon to this mechanism is the overlap of *a*_1g_ and *e*_g_^π^ bands due to the modulations of Ti–Ti distances along the *c*-axis of the crystal lattice^5,8^. Owing to the trigonal distortions in octahedral geometry, the *t*_2g_ levels in TiO_6_ octahedra further split into *a*_1g_ and *e*_g_^π^ levels. The *a*_1g_ orbitals between the face-shared TiO_6_ octahedra along the *c*-axis are strongly hybridized to form *a*_1g_ and *a*_1g_* bands with *e*_g_^π^ bands between them. When Ti–Ti bond distances along the *c*-axis are short, the energy splitting between *a*_1g_ and *a*_1g_* bands becomes large so that the *e*_g_^π^ bands do not overlap with the *a*_1g_ bands. Furthermore, only the *a*_1g_ band is completely filled with Ti 3*d* electrons; therefore, Ti_2_O_3_ acts as an insulator.

The energy diagram describes the electronic structures of Ti_2_O_3_ revealing the close connection between the MIT and *c*/*a* ratio. The *a* and *c* lattice constants of Ti_2_O_3_ vary significantly with temperatures, and the *c*/*a* ratio increases from 2.648 at 373 K to 2.701 at 553 K across the MIT^4^. The modulation of Ti 3*d* electron occupations in *a*_1g_ orbitals associated with a change in the *c*/*a* ratio was revealed from the temperature dependence of linear dichroism in Ti 2*p* X-ray absorption spectra^3,20,21^. However, the band-structure calculations concerned the validity of this simple phenomenological model because *a*_1g_ and *e*_g_^π^ bands always overlap for typical Ti–Ti distances. A short Ti–Ti distance of less than 2.2 Å is required to form the aforementioned insulating band diagram^14^, thereby confirming the importance of electron correlations in Ti_2_O_3_. Therefore, to understand the mechanism of this MIT better, studies on physical properties of Ti_2_O_3_ as a function of *c*/*a* ratios are required.

Recently, single-crystalline corundum-type Ti_2_O_3_ films were synthesized on isostructural *α*-Al_2_O_3_ (0001) substrates^23–25^, wherein their physical properties were found modulated from those of the bulk systems due to the lattice deformations. The MIT temperature was found in the range of ~ 200–300 K for the films, which is ~ 200 K lower than that of the bulk^23,24^. Besides, a significant increase in the *c* lattice constant was detected in the films (at RT *c* ~ 13.8 Å in the films and *c* = 13.61 Å in the bulk). Considering the close relationship between the *c*/*a* ratio and MIT observed in the bulk system, it can be presumed that the suppression of the insulating states in the films is equally related to the lattice deformations. Unfortunately, lattice deformations are not expected in the films grown coherently on *α*-Al_2_O_3_ (0001) substrates due to the large lattice mismatch between *α*-Al_2_O_3_ and Ti_2_O_3_ (~ 8.3%). Furthermore, in contrast to the perovskite-type oxides, corundum-type oxides have the only option of *α*-Al_2_O_3_ as an isostructural substrate, which makes it difficult to investigate the physical properties as a function of the *c*/*a* ratio by conventional epitaxial-strain manipulations.

To investigate the relationship between *c*/*a* ratios and MIT in Ti_2_O_3_, we demonstrate another approach to control the lattice deformation in Ti_2_O_3_ films, which involves different growth temperatures to manipulate domain sizes and thereby different *c*/*a* ratios. The substrate temperature plays a crucial role in the nucleation of films, and domain sizes vary depending on the growth conditions^26^. In corundum-type oxides such as Ti_2_O_3_ and V_2_O_3_, it is known that *a* and *c* lattice constants evolve with the size of nanoparticles^15,27^. Therefore, Ti_2_O_3_ films grown under different temperatures are expected to render different *a* and *c* lattice constants and electrical properties. In this study, we have grown corundum-type Ti_2_O_3_ films at high (1000 ºC) and low (500 ºC) temperatures. The successful synthesis of corundum-type Ti_2_O_3_ films on *α*-Al_2_O_3_ substrates at different temperatures was confirmed by Raman spectroscopy and X-ray diffraction (XRD) experiments. The *c*/*a* ratios at RT were determined as 2.696 and 2.781 for the films grown at high and low temperatures, respectively. Notably, metallic ground states appeared for the film grown under low temperatures, manifesting a large *c*/*a* ratio with domain sizes of the order of 10 nm, whereas MIT was observed in the film grown at high temperatures. We discuss the origin of this MIT behavior in the Ti_2_O_3_ films by adopting density functional theory (DFT)-based electronic-structure calculations.

## Results and discussion

**Figure 1:**
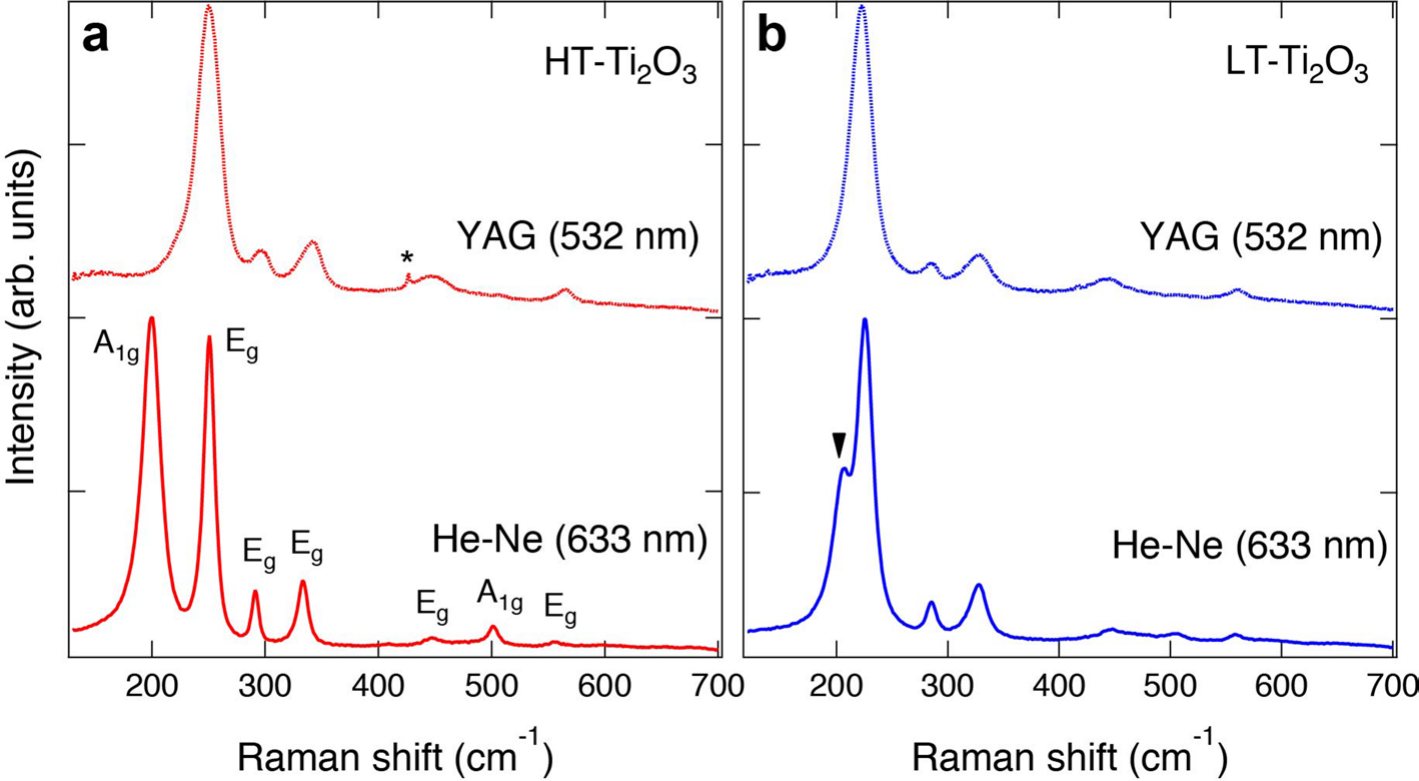
Investigation of corundum-type crystal structure in the Ti_2_O_3_ films. Raman spectra of (**a**) HT- and (**b**) LT-Ti_2_O_3_ films measured at RT. The spectra were collected using YAG (dotted lines) and He–Ne (solid lines) lasers. Vibrational modes (*A*_1g_ or *E*_g_) are indicated in the spectrum of the HT-Ti_2_O_3_ film recorded using He–Ne laser. The triangle in (**b**) indicates the peak related to the low-frequency *A*_1g_ mode. The asterisk indicates the peak from the *α*-Al_2_O_3_ substrate.

Figure 1 shows the Raman spectra of the high-temperature-Ti_2_O_3_ (HT-Ti_2_O_3_) and low-temperature-Ti_2_O_3_ (LT-Ti_2_O_3_) films. The Raman spectrum of the HT-Ti_2_O_3_ film recorded using a He–Ne laser shows seven peaks, which is consistent with previous reports^11–13,24,25^. In corundum-type crystal structures, two *A*_1g_ and five *E*_g_ modes are Raman active following the symmetry considerations^28^. To reveal the vibrational modes in the HT-Ti_2_O_3_ film, we further performed Raman spectroscopy using another light source of a Nd:Y_3_Al_5_O_12_ (YAG) laser, as shown in Fig. 1a. Evidently, the lowest-frequency mode was not observed in the spectrum due to the resonant Raman effect reflecting the electronic structures of corundum-type Ti_2_O_3_^13^. Therefore, the 1st and 2nd low-frequency modes were assigned to the *A*_1g_ and *E*_g_ modes, respectively, which are consistent with the earlier report by Shin et al*.*^12,13^ (see Supplementary Note 1). Our results indicate that both peak structures and resonant Raman effects are good fingerprints to elucidate corundum-type Ti_2_O_3_^11–13,24,25,29–34^.

Similar Raman spectra were obtained for the LT-Ti_2_O_3_ film, as shown in Fig. 1b, confirming the same crystal structure as that of the HT-Ti_2_O_3_ film. The Raman spectrum recorded using the He–Ne laser also shows seven peaks, although the peak shapes were slightly different from those of the HT-Ti_2_O_3_ film. The *E*_g_ modes softened, and in contrast, the frequency of the low-frequency *A*_1g_ mode increased, as shown by the filled triangle in Fig. 1b. Therefore, the *A*_1g_ and *E*_g_ modes at ~ 230 cm^−1^ were overlapped with each other. These behaviors were also observed in the bulk systems at high temperatures rendering large *c*/*a* ratios^12^. We will discuss the difference in the Raman spectra between the HT- and LT-Ti_2_O_3_ films later in connection with the transport properties. Nevertheless, the Raman spectrum of the LT-Ti_2_O_3_ film measured using the YAG laser was in good agreement with that of the HT-Ti_2_O_3_ film, as shown in Fig. 1b. These results suggest that the LT-Ti_2_O_3_ film exhibits corundum-type crystal structures and its electronic structures are similar to those in bulk Ti_2_O_3_ at high temperatures.

**Figure 2:**
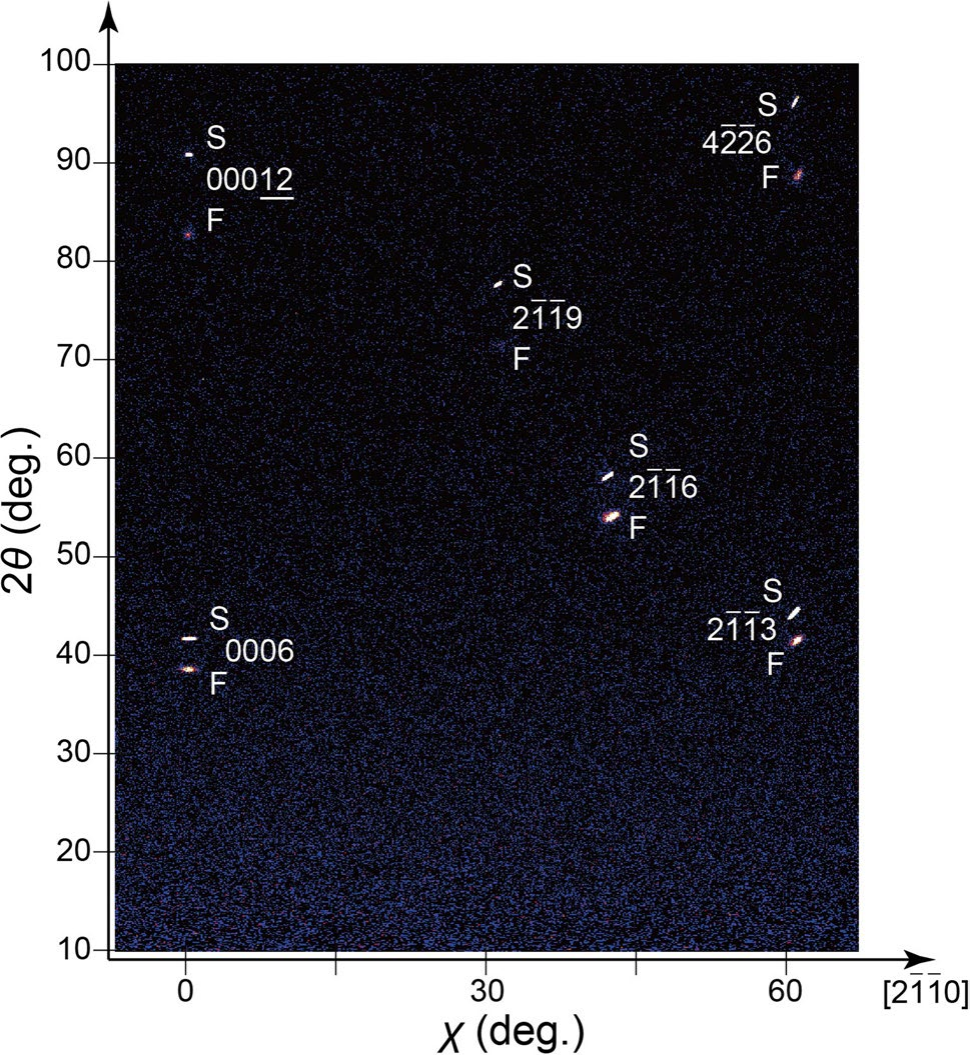
Identical crystal structure for the LT-Ti_2_O_3_ film and *α*-Al_2_O_3_ substrate. 2D XRD contour map of the LT-Ti_2_O_3_ film on *α*-Al_2_O_3_ (0001) substrates. The labels F and S denote the reflections from the LT-Ti_2_O_3_ film and *α*-Al_2_O_3_ substrate, respectively. The tilt angle *χ* = 0° corresponds to out-of-plane directions. The azimuthal angle *φ* is fixed along [2-1-10] direction.

Corundum-type crystal structure of the LT-Ti_2_O_3_ film was further confirmed from the two-dimensional (2D) XRD contour map, as shown in Fig. 2. At the tilt angle *χ* = 0º, which corresponds to out-of-plane direction, *α*-Al_2_O_3_ 0006 and 00,012 reflections were detected at 2*θ* =  ~ 42º and 91º, respectively. The reflections from the film were detected at 2*θ* =  ~ 38º and 84º at the tilt angle *χ* = 0º, which can be assigned to 0006 and 00012 reflections of Ti_2_O_3_, respectively. All the film reflections were detected at lower 2*θ* angles than the substrate reflections at the same tilt angle *χ*. Such pairs of film and substrate reflections observed from the 2D XRD contour maps confirm the identical crystal symmetry of the film and substrate. The 2D XRD contour maps taken along other in-plane directions are shown in Supplementary Fig. 1. These results confirm that the LT-Ti_2_O_3_ film also exhibits corundum-type crystal structure. Both in-plane and out-of-plane orientations were identical in the LT-Ti_2_O_3_ film and *α*-Al_2_O_3_ substrates.

**Figure 3:**
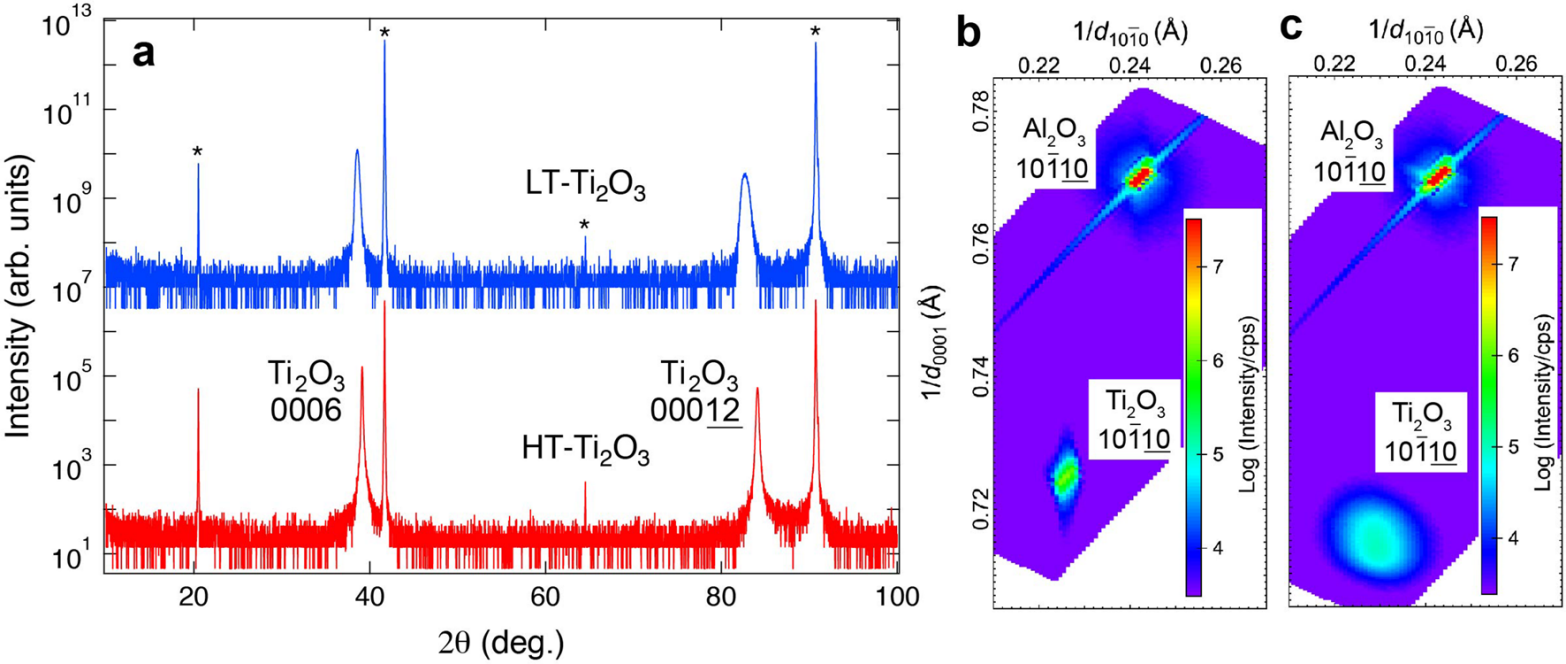
Determination of in-plane and out-of-plane lattice constants of the Ti_2_O_3_ films. (**a**) Out-of-plane XRD patterns for the HT- and LT-Ti_2_O_3_ films. The asterisks indicate the reflection coming from *α*-Al_2_O_3_ (0001) substrates. Reciprocal space maps around *α*-Al_2_O_3_ 10-110 reciprocal points in the cases of the (**b**) HT- and (**c**) LT-Ti_2_O_3_ films.

The detailed lattice constants of the Ti_2_O_3_ films were determined from the XRD measurements. Figure 3a shows the out-of-plane XRD patterns from the HT- and LT-Ti_2_O_3_ films. Herein, 0006 and 00012 reflections of the HT-Ti_2_O_3_ (LT-Ti_2_O_3_) film were detected at 2*θ* = 39.13º and 84.11º (38.57º and 82.66º), respectively. The *c* lattice constant of the HT-Ti_2_O_3_ (LT-Ti_2_O_3_) film was 13.80 Å (14.00 Å), which is larger than that of the bulk Ti_2_O_3_ at RT (*c* = 13.61 Å)^4^. The possible origin of the increased *c* lattice constants can be smaller domain sizes, which will be discussed later. The in-plane lattice constants were determined from the reciprocal space maps. Figure 3b,c shows the reciprocal space maps of the HT- and LT-Ti_2_O_3_ films around the *α*-Al_2_O_3_ 10-110 reciprocal point, respectively. From the reciprocal point of Ti_2_O_3_ 10-110, *a* lattice constant of the HT-Ti_2_O_3_ (LT-Ti_2_O_3_) film was found 5.119 Å (5.034 Å). The resultant *c*/*a* ratio of the HT-Ti_2_O_3_ (LT-Ti_2_O_3_) film was 2.696 (2.781), which is much larger than that of the bulk Ti_2_O_3_ (*c*/*a* = 2.639). Note that the *c*/*a* ratio of the LT-Ti_2_O_3_ film is also much larger than that of typical corundum-type oxides such as α-Al_2_O_3_ (*c*/*a* = 2.732 where *a* = 4.759 Å and *c* = 12.99 Å), but relatively smaller than that of another corundum-type oxide V_2_O_3_ (*c*/*a* = 2.828 where *a* = 4.9717 Å and *c* = 14.005 Å)^35^.

**Figure 4:**
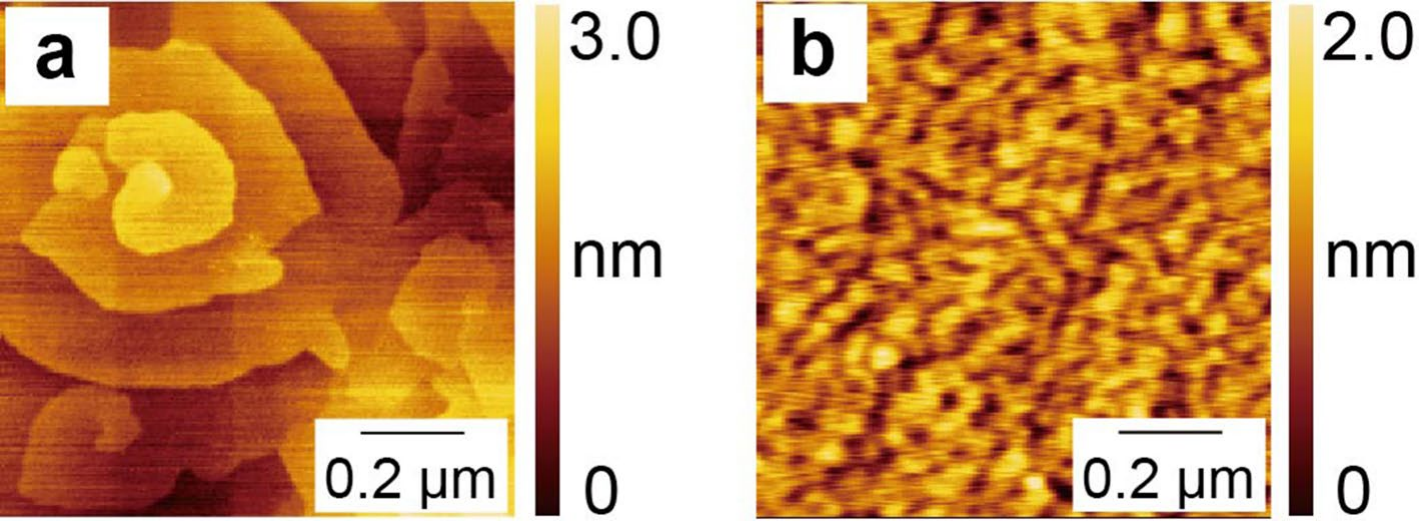
Surface morphology of the Ti_2_O_3_ films. AFM images of the (**a**) HT- and (**b**) LT-Ti_2_O_3_ films.

The different growth temperatures also significantly affected the surface morphologies of the films. As shown in Fig. 4a, the atomic force microscope (AFM) image of the HT-Ti_2_O_3_ film reflected a trace of spiral growths^36^, which was also reported previously^23^. The in-plane size of the spirals was ~ 0.5 µm. In contrast, the LT-Ti_2_O_3_ film did not exhibit such spiral growths in the AFM image as shown in Fig. 4b. Instead, smaller grains of less than 50 nm were detected at the surface, which can be attributed to the reduced migration energies during the low-temperature growth process^26^. Out-of-plane grain sizes were roughly estimated from the out-of-plane XRD patterns (See Supplementary Note 2). The broad peaks in the LT-Ti_2_O_3_ film, as shown in Fig. 3a, also suggested small grain sizes. Grain sizes of the LT-Ti_2_O_3_ film were estimated as 23 nm from the Scherrer equation, which is comparable to the estimation from the AFM image (See Supplementary Note 2 for the detailed analyses). In corundum-type Ti_2_O_3_ and V_2_O_3_ nanoparticles, it was reported that lattice constants evolved greatly with the particle sizes^15,27^. In the case of Ti_2_O_3_, the *c* (*a*) lattice constant becomes longer (shorter) as the grain size decreases, resulting in an increased *c*/*a* ratio for the nanoparticles^15^. Considering the larger size (~ 1 mm scale) of the Ti_2_O_3_ single crystal, the negative correlation between the domain size and *c*/*a* ratio is plausible in the films.

**Figure 5:**
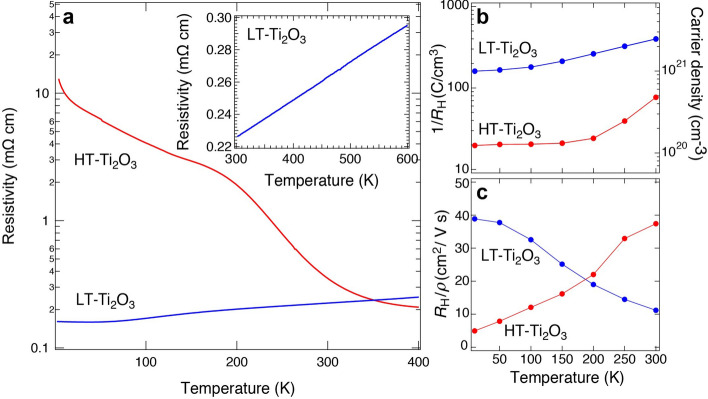
Electric properties of the Ti_2_O_3_ films. (**a**) Temperature dependence of resistivity for the HT- and LT-Ti_2_O_3_ films. The inset shows temperature dependence of resistivity for the LT-Ti_2_O_3_ film above RT. Temperature dependence of the (**b**) inverse Hall coefficient 1/*R*_H_ and (**c**) Hall mobility *R*_H_/*ρ* of the HT- and LT-Ti_2_O_3_ films.

It is well established that the *c*/*a* ratio is strongly related to the MIT in the bulk Ti_2_O_3_^4^. To elucidate the relationship between the MIT and *c*/*a* ratios in the Ti_2_O_3_ films, we performed temperature-dependent resistivity measurements for the HT- and LT-Ti_2_O_3_ films, as shown in Fig. 5a. For the HT-Ti_2_O_3_ film, the resistivity at 2 K was ~ 10 mΩ cm. The resistivity gradually decreased with increasing temperatures and the broad MIT appeared at 200–300 K accompanying a substantial change in the resistivity. Even beyond the MIT, d*ρ*/dT remained negative up to 400 K (*ρ* and T denote resistivity and temperature, respectively). Such resistivity behaviors suggest the suppression of insulating states in Ti_2_O_3_ films in comparison with bulk, which is also consistent with the previous reports^23,24^. In bulk, the resistivity at ~ 10 K is more than 10^2^ Ω cm^2,3^, which is four orders of magnitude larger than that of the HT-Ti_2_O_3_ film. In addition, the MIT temperature in bulk is ~ 450 K, which is ~ 200 K higher than that of the film^23,24^.

Suppression of the insulating states is further examined in the LT-Ti_2_O_3_ film that show a completely different resistivity curve, as shown in Fig. 5a. The MIT was not observed and metallic conductivity (d*ρ*/dT > 0) appeared until ~ 50 K. However, at temperatures < 50 K, a slight upturn of the resistivity curve was noticed, suggesting a trap of conductive carriers in defects at low temperatures, which can be related to the poor crystallinity of the LT-Ti_2_O_3_ film (See Supplementary Fig. 2). In bulk (Ti_1−*x*_V_*x*_)_2_O_3_, resistivity of low-temperature insulating phases decrease drastically with V doping, but another phase transition was detected at high-temperature regions (~ 450 K)^2^. To eliminate the possible phase transition in the LT-Ti_2_O_3_ film, we further performed high-temperature resistivity measurements, as shown in the inset of Fig. 5a. The metallic conductivity continued up to 600 K with a linear *ρ*–T relationship. We note that metallic conductivity first appears at ~ 500 K for the HT-Ti_2_O_3_ film^23^, but it is never observed for a bulk system up to 575 K^2,3^.

The electric properties were further investigated from Hall-effect measurements. The clear linear dependence of the Hall resistance on the magnetic field was observed for both films (see Supplementary Fig. 3). We plot the resultant inverse Hall coefficient (1/*R*_H_) in Fig. 5b as a function of temperature. The positive 1/*R*_H_ indicates the hole-carrier conduction of both films, which is consistent with the previous reports^1,37^. The 1/*R*_H_ became smaller with decreasing temperature for both films. In particular, the 1/*R*_H_ of the HT-Ti_2_O_3_ film drastically decreased in the temperature range from 300 to 200 K, reflecting the occurrence of MIT. Assuming the single-band model, we estimated the carrier densities of the films as indicated by the scale on the right axis in Fig. 5b. The carrier densities at 300 K were 4.8 × 10^20^ cm^−3^ and 2.5 × 10^21^ cm^−3^ for the HT- and LT-Ti_2_O_3_ films, respectively. The temperature dependence of the Hall mobility (*R*_H_/*ρ*) was also plotted in Fig. 5c. The *R*_H_/*ρ* decreased to be < 10 cm^2^/V s in low temperatures for the HT-Ti_2_O_3_ film. In contrast, the *R*_H_/*ρ* was as high as 10–40 cm^2^/V s for the LT-Ti_2_O_3_ film in the whole temperatures, supporting the metallic ground states of the LT-Ti_2_O_3_ film.

The metallic states of the LT-Ti_2_O_3_ film are further supported by Raman spectroscopy (Fig. 1). Shin et al. reported that the low-frequency A_1g_ mode is an excellent indicator of the electrical behaviors of Ti_2_O_3_^12,13^. The A_1g_ vibrational modes correspond to breathing against one another for the Ti atom pairs along the *c*-axis. Simultaneously, oxygen atoms forming triangles between each Ti pair are moving in and out alternately with respect to each other. In the low-frequency A_1g_ mode, oxygen atoms move out when the Ti atoms move in, so that the screening effect of the oxygens becomes lowest, and the modulation of the trigonal component to the crystalline field rises to its largest value. In a phenomenological model, the intensity of Raman mode is related to the polarizability of the electron cloud. As shown in Fig. 1a, the Raman spectrum of the HT-Ti_2_O_3_ film collected using the He–Ne laser showed the strongest intensity for the low-frequency A_1g_ mode, which is consistent with the phenomenological model. In contrast, the intensity of the A_1g_ mode was strongly suppressed in the LT-Ti_2_O_3_ film due to conduction-carrier screening of the electric field caused by the electron cloud motions. Since the screening effect from the oxygens is weakest in the low-frequency A_1g_ mode, its intensity gets most affected. Therefore, the strong suppression of the low-frequency A_1g_ mode provides further evidence for the metallic conductivity in the LT-Ti_2_O_3_ film.

One might suspect that different carrier densities (4.8 × 10^20^ cm^−3^ for the HT-Ti_2_O_3_ film and 2.5 × 10^21^ cm^−3^ for the LT-Ti_2_O_3_ film at 300 K) are caused by extrinsic effects such as oxygen non-stoichiometry. However, it is unlikely in the present case. In Ti_2_O_3_, the atomic density of Ti in the crystal lattice is calculated to be 3.8 × 10^22^ cm^–3^. The carrier densities correspond to be 1.3 and 6.6% holes /Ti for the HT- and LT-Ti_2_O_3_ films, respectively. Meanwhile, Andersson et al*.* revealed that the corundum-type Ti_2_O_3_ phase was only stable with the oxygen non-stoichiometry of less than 1%, corresponding to the chemical formula of Ti_2_O_2.98_–Ti_2_O_3.02_^38^. These results suggest that the carrier density of the LT-Ti_2_O_3_ film is not explained by the oxygen non-stoichiometry.

The plausible scenario to explain different hole-carrier densities between the HT- and LT-Ti_2_O_3_ films is the intrinsic holes whose densities are varied by the *c*/*a* ratio. Chang et al. revealed the change of the Ti 3*d* electron occupation in the *a*_1g_ and *e*_g_^π^ orbitals by the *c*/*a* ratio^3^. We note that the *c*/*a* ratio directly corresponds to the degree of the trigonal distortion in TiO_6_ octahedra and the distance of Ti–Ti pair along the *c*-axis. When the *c*/*a* ratio was small enough, the Ti 3*d* electrons completely filled the most stable *a*_1g_ orbital, resulting in the formation of 100% *a*_1g_*a*_1g_ singlet state in the Ti–Ti pair. With increasing the *c*/*a* ratio, the occupation in the *a*_1g_ orbital systematically decreased. According to Ref. 3, the *a*_1g_*a*_1g_ singlet state was reduced to be 49% at 575 K (*c*/*a* = 2.70). The reduction in the *a*_1g_*a*_1g_ singlet state is responsible for increasing the hole carriers in the *a*_1g_ bands. Moreover, the rest of the Ti 3*d* electrons is partially filled with the *e*_g_^π^ orbital that expands in the *a*–*b* plane. The partially-filled *e*_g_^π^ orbital forms conducting paths to the neighboring Ti–Ti pairs, resulting in the large hole-carrier densities and metallic conductivity in the LT-Ti_2_O_3_ film.

**Figure 6:**
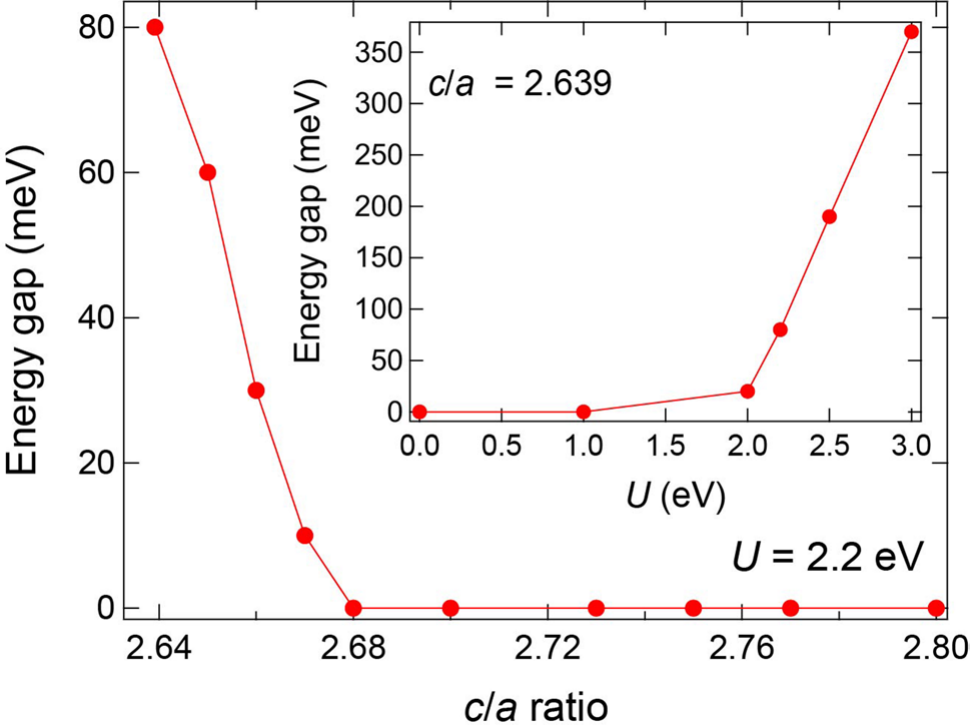
Lattice deformation induced MIT from DFT calculation. Plots of the energy gap at *E*_F_ from DFT + *U* calculations with *U* = 2.2 eV as a function of *c*/*a* ratios. The inset shows plots of the energy gap at *E*_F_ in bulk Ti_2_O_3_ (*c*/*a* = 2.639) as a function of *U* values.

Next, to reveal the origin of different electronic states in the films and bulk materials, we performed DFT + *U* calculations using the Quantum ESPESSO simulation software^39,40^. Since insulating ground states were not reproduced in Ti_2_O_3_ by DFT calculations^14^, we first estimated adequate Hubbard *U* parameter to reproduce the experimental energy gap. The inset of Fig. 6 shows the energy gap at the Fermi level (*E*_F_) of Ti_2_O_3_ with a *c*/*a* ratio of 2.639 (bulk value at RT) as a function of *U* values. When *U* was not introduced in calculations, the energy gap was 0 meV, corresponding to the metallic ground states. The energy gap emerged when *U* was larger than 2.0 eV, and it gradually increased with an increase in *U* values. For example, the energy gaps at *U* = 2.2, 2.5, and 3.0 eV were 80, 190, and 370 meV, respectively. Considering the experimental energy gap of ~ 100 meV^1^, we set *U* = 2.2 eV for the subsequent calculations (See Supplementary Fig. 4 for the DOS of Ti_2_O_3_).

The MIT derived from the lattice deformations was demonstrated by the DFT + *U* calculations. Figure 6 shows the energy gap at *E*_F_ as a function of *c*/*a* ratios at *U* = 2.2 eV. The energy gap was 80 meV at *c*/*a* = 2.639, and it became narrower with an increase in the *c*/*a* ratio. Eventually, the gap closed at a critical *c*/*a* ratio of 2.68, whereas the metallic ground states continued to be stable up to a *c*/*a* ratio of 2.8. The critical *c*/*a* ratio of 2.68 is in good agreement with the experimental results^4^. In bulk materials, the *c*/*a* ratio reaches 2.68 at ~ 470 K in the middle of the broad MIT. Considering the *c*/*a* ratios of the HT-Ti_2_O_3_ (2.696) and LT-Ti_2_O_3_ (2.781) films, both types of films were in metallic regions at RT, corroborating the *ρ*–T curves provided in Fig. 5a.

We will discuss the plausible origin behind the occurrence of the MIT in the HT-Ti_2_O_3_ film and the absence of the MIT in the LT-Ti_2_O_3_ film at low temperatures. The *a* and *c* lattice constants should be modulated as a function of temperature due to the thermal expansion. To experimentally estimate thermal expansion coefficients (α) of the Ti_2_O_3_ films, we performed the temperature-dependent XRD measurements (see Supplementary Figs. 6–8). The α along the *c*-axis (*α*_c_) and *a*-axis (*α*_a_) were estimated to be *α*_c_ = 1.86 ± 0.17 × 10^–5^ K^−1^ and *α*_a_ = 2.10 ± 2.97 × 10^–6^ K^−1^ for the HT-Ti_2_O_3_ film and to be *α*_c_ = 1.70 ± 0.11 × 10^–5^ K^−1^ and *α*_a_ = − 0.37 ± 3.32 × 10^–6^ K^−1^ for the LT-Ti_2_O_3_ film. The relative errors of the *α*_a_ look large, which originates temperature-independent *a* lattice constants. We also referred to the α of bulk Ti_2_O_3_ that was estimated to be α_c_ = 3.263 × 10^–5^ K^−1^ and α_a_ = − 1.088 × 10^–6^ K^−1^ using previous results (see Supplementary Fig. 9)^4^. We found that the *α*_c_ of the films was approximately half of the *α*_c_ of the bulk specimen. We utilized the α determined from the present experiments and the bulk reference and then obtained the *c*/*a* ratios of the films below RT by the linear extrapolation of the values in the high-temperature metallic region to low temperatures (See Supplementary Fig. 10). In the HT-Ti_2_O_3_ film, the *c*/*a* ratio reached the critical value of 2.68 at ~ 150 K using the α of the bulk reference and at ~ 50 K using the experimentally determined α. Although these critical MIT temperatures are much lower than that revealed from the temperature dependence of resistivity (~ 250 K in Fig. 5a), the results qualitatively demonstrate that the MIT in the HT-Ti_2_O_3_ film is due to the temperature dependence of the crystal deformation. In contrast, the LT-Ti_2_O_3_ film did not reach the critical *c*/*a* ratio of 2.68 even at the lowest temperature limit. The estimated *c*/*a* ratio at 2 K was larger than 2.754, where the metallic states appeared in the DFT calculations and in experiments for the bulk materials. These results suggest that the electrical properties of both HT- and LT-Ti_2_O_3_ films can be explained by the *c*/*a* ratio as in the bulk systems. Absence of the MIT in the LT-Ti_2_O_3_ film can be attributed to the elongated *c* lattice constant (at RT *c* = 14.00 Å in the film and *c* = 13.61 Å in the bulk) caused by the nano-sized domains.

We presume that treatment of the empirical parameter *U* is responsible for the quantitative discrepancies in the critical temperatures between the experiment and the calculation. In the DFT + *U* calculations, the critical *c*/*a* ratio had a positive correlation with *U*. Moreover, the energy gap quite sensitively depended on *U*, as shown in Fig. 6. In this study, we merely selected *U* = 2.2 eV to reproduce the energy gap (~ 100 meV) for bulk Ti_2_O_3_. Therefore, it is quite difficult to determine the critical *c*/*a* ratio from the DFT + *U* calculations quantitatively at the moment, although the close relationship between *c*/*a* ratios and MIT in Ti_2_O_3_ is clearly demonstrated on the qualitative level. To resolve the quantitative discrepancies between the experiments and calculations, further experimental studies to determine the energy gap of bulk Ti_2_O_3_ precisely and more accurate theoretical studies would be required.

Herein, we have grown Ti_2_O_3_ films on *α*-Al_2_O_3_ (0001) substrates at high and low temperatures to control domain sizes and *c*/*a* ratios of the structure. Successful synthesis of films exhibiting corundum-type crystal structure in both conditions was confirmed by Raman spectroscopy and XRD measurements. The *c*/*a* ratios at RT were significantly different in the HT-Ti_2_O_3_ (2.696) and LT-Ti_2_O_3_ (2.781) films. The HT-Ti_2_O_3_ film showed the broad MIT at ~ 200–300 K, which was ~ 200 K lower than that of the bulk. However, the LT-Ti_2_O_3_ film exhibited metallic conductivity and did not show any MIT up to 600 K. The DFT + *U* calculations predicted the critical *c*/*a* ratio of 2.68 to induce the MIT. The detailed analyses revealed that the observed electrical properties of the Ti_2_O_3_ films can be explained by the critical *c*/*a* ratio and anisotropic thermal expansion coefficients. These results suggest that the electrical properties of Ti_2_O_3_ films are also governed by the *c*/*a* ratio of the structure and the absence of the MIT in the LT-Ti_2_O_3_ film is due to the lattice deformations characterized by an increased *c* lattice constant leading to an enhanced *c*/*a* = 2.781 at RT.

## Methods

### Thin-film growth

Ti_2_O_3_ films were grown on *α*-Al_2_O_3_ (0001) substrates using the pulsed-laser deposition (PLD) method. Polycrystalline ceramics of TiO (3 N purity, purchased from Toshima Manufacturing Co., Ltd.) were used as the PLD target. A KrF excimer laser (0.8 J/cm^2^, 5 Hz) was used for target ablations. The substrate temperature and oxygen partial pressure were set 1000 ºC and 1.5 × 10^–6^ Torr, respectively, for the production of the HT-Ti_2_O_3_ film, whereas those for the preparation of the LT-Ti_2_O_3_ film with single-phases were 500 ºC and 5.0 × 10^–7^ Torr, respectively. Notably, when the temperature was set between 500 and 1000 °C, another superconducting-titanate phase was stabilized and consequently, single-phase corundum-type Ti_2_O_3_ films were not obtained (See Supplementary Figs. 11–14). At the end of the growth process, the oxygen flow to the PLD chamber was stopped immediately and films were quenched to RT to avoid the occurrence of additional oxidations^23,41^. Film thickness (~ 100 nm) was measured using a stylus-type profiler.

### Thin-film characterizations

Crystal structures of the films were revealed by Raman spectroscopy and XRD measurements conducted at RT. Raman spectra were acquired using a LabRAM HR-800 (Horiba) equipped with YAG (*λ* = 532 nm) and He–Ne (*λ* = 633 nm) lasers. The laser incident and Raman scattering directions were parallel to the *c*-axis. The polarization vector of the lasers was set parallel to the Ti_2_O_3_ [10-10] direction. XRD patterns and contour maps were measured using the Cu–K*α*_1_ radiation available in a SmartLab 9 kW diffractometer (Rigaku). Surface morphology was examined via AFM. Temperature dependence of resistivity was measured by implementing a standard four-probe method using PPMS (Quantum Design) at low temperatures (2 ≤ T ≤ 400 K) and PPHL-800 (Pascal) at high temperatures (300 ≤ T ≤ 600 K). Au electrodes were deposited by the sputtering method. Ohmic contacts between the films and electrodes were confirmed from the two probe I–V measurements at RT. Hall-effect measurements were carried out in a Hall-bar geometry using PPMS.

### Electronic structure calculations

DFT calculations were performed using the Quantum ESPRESSO simulation software^39,40^. Ultrasoft pseudopotentials were used, wherein atomic Ti 3*s*, 3*p*, 3*d*, and 4*s*, O 2*s*, and 2*p* levels were included as valence-band states. The Perdew–Burke–Ernzerhof generalized gradient approximation (PBE-GGA) function was utilized for the exchange–correlation potentials^42^. The kinetic energy (charge density) cut-off was set as 60 Ry (600 Ry). Ti and O positions were optimized by a structural relaxation routine implementing Monkhorst–Pack scheme with a 6 × 6 × 6 k-mesh in self-consistent calculations^43^. Accuracy in total energy after the convergence of the self-consistent calculations was less than 10^–10^ Ry. After the structural optimization, the density of states (DOS) was calculated in non-self-consistent fields using a denser 12 × 12 × 12 k-mesh. The tetrahedron method was applied to integrate the DOS in the Brillouin zone^44^. Further computing details are described in Supplementary Methods.

## Supplementary Information

Supplementary Information.

## Data Availability

The data that support the findings of this study are available from the corresponding author upon reasonable request.
