# Supplementary material for: Metallic ground states of undoped Ti2O3 films induced by elongated c-axis lattice constant
Source: Sci Rep. 2020 Dec 17;10:22109. doi: 10.1038/s41598-020-79182-5 (PMC7747712; doi:10.1038/s41598-020-79182-5)
Supplement: Supplementary file 1 — Supplementary Information. [file 41598_2020_79182_MOESM1_ESM.docx]

**Supplemental Information**

**Metallic ground states of undoped Ti_2_O_3_ films induced by elongated *c*-axis lattice constant**

K. Yoshimatsu^1,2^, N. Hasegawa^1^, Y. Nambu^3^, Y. Ishii^4^, Y. Wakabayashi^2,4^, and H. Kumigashira^1,2,5^

^1^Institute of Multidisciplinary Research for Advanced Materials, Tohoku University Sendai 980-8577, Miyagi, Japan

*^2^Materials Research Center for Element Strategy (MCES), Tokyo Institute of Technology, Yokohama 226-8503, Japan*

*^3^Institute of Materials Research, Tohoku University Sendai 980-8577, Miyagi, Japan*

*^4^Department of Physics, Tohoku University, Sendai 980-8578 Miyagi, Japan*

*^5^Photon Factory, Institute of Materials Structure Science, High Energy Accelerator Research Organization (KEK), 1-1 Oho, Tsukuba 305-0801, Japan*

Author to whom correspondence should be addressed: kohei.yoshimatsu.c6@tohoku.ac.jp.

2D XRD contour maps along various in-plane directions

Supplementary Figure 1 shows 2D XRD contour maps of the LT-Ti_2_O_3_ film along various in-plane directions. Along each direction, several pairs of film and substrate reflections at the same tilt angles *χ* were observed, which confirmed the manifestations of identical crystal symmetry from the film and the substrate.


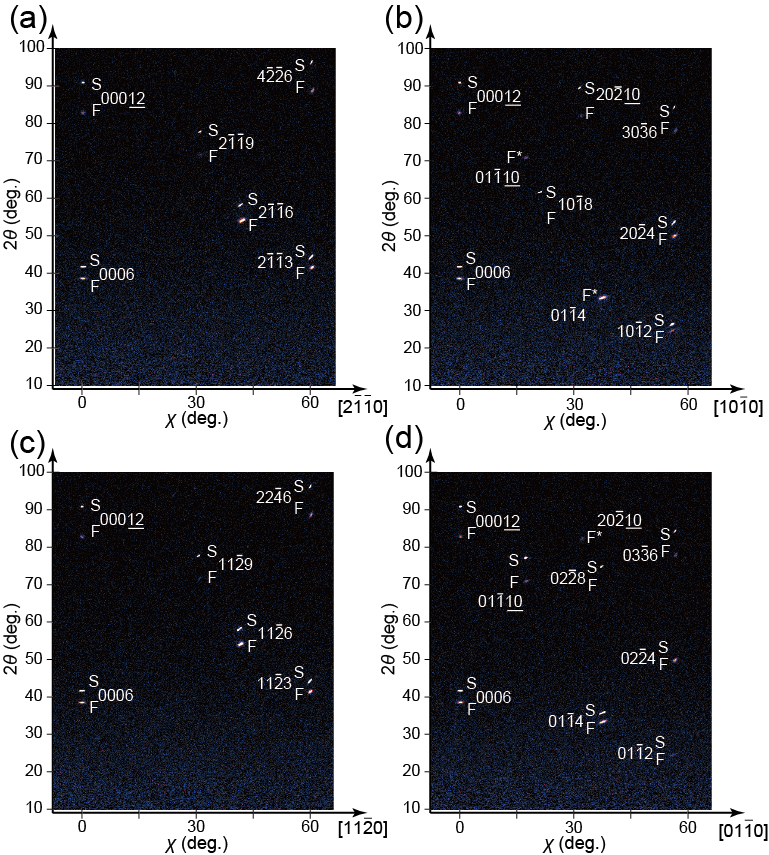


**Supplementary Figure 1. 2D XRD contour maps of the LT-Ti_2_O_3_ film.** The contour maps were taken along (a) [2-1-10], (b) [10-10], (c) [11-20], and (d) [01-10] directions. The labels F and S denote the reflections from the LT-Ti_2_O_3_ film and *α*-Al_2_O_3_ substrate, respectively. The label F* denotes the reflections from the 180° rotational domains in the LT-Ti_2_O_3_ film.

Crystallinity of HT- and LT-Ti_2_O_3_ films

Supplementary Figure 2 shows the X-ray rocking curve profiles of 0006 reflections for the HT- and LT-Ti_2_O_3_ films. Full widths at half maximum (FWHMs) of the reflections were 0.06° and 0.56° for the HT- and LT-Ti_2_O_3_ films, respectively. High crystallinity of the HT-Ti_2_O_3_ film was revealed from the X-ray rocking curve. In contrast, the LT-Ti_2_O_3_ film exhibits poor crystallinity due to the lower growth temperature.

**Supplementary Figure 2. X-ray rocking curve profiles of Ti_2_O_3_ 0006 reflection.** The red and blue colors indicate for the HT-Ti_2_O_3_ and LT-Ti_2_O_3_ films, respectively.

Temperature dependence of Hall resistance for HT- and LT-Ti_2_O_3_ films

Supplementary Figure 3 shows the magnetic-field dependence of the Hall resistance (*R_xy_*) for the Ti_2_O_3_ films at various temperatures. The linear *R_xy_* with the positive slopes for both films indicates their hole-carrier conduction, which is consistent with the previous reports^[1, 33]^. The slopes of the LT-Ti_2_O_3_ film were smaller by an order of magnitude than those of the HT-Ti_2_O_3_ film, suggesting larger carrier density in the LT-Ti_2_O_3_ film. The carrier densities at 300 K estimated on the basis of the single-band model were 4.8 × 10^20^ cm^-3^ and 2.5 × 10^21^ cm^-3^ for the HT- and LT-Ti_2_O_3_ films, respectively.


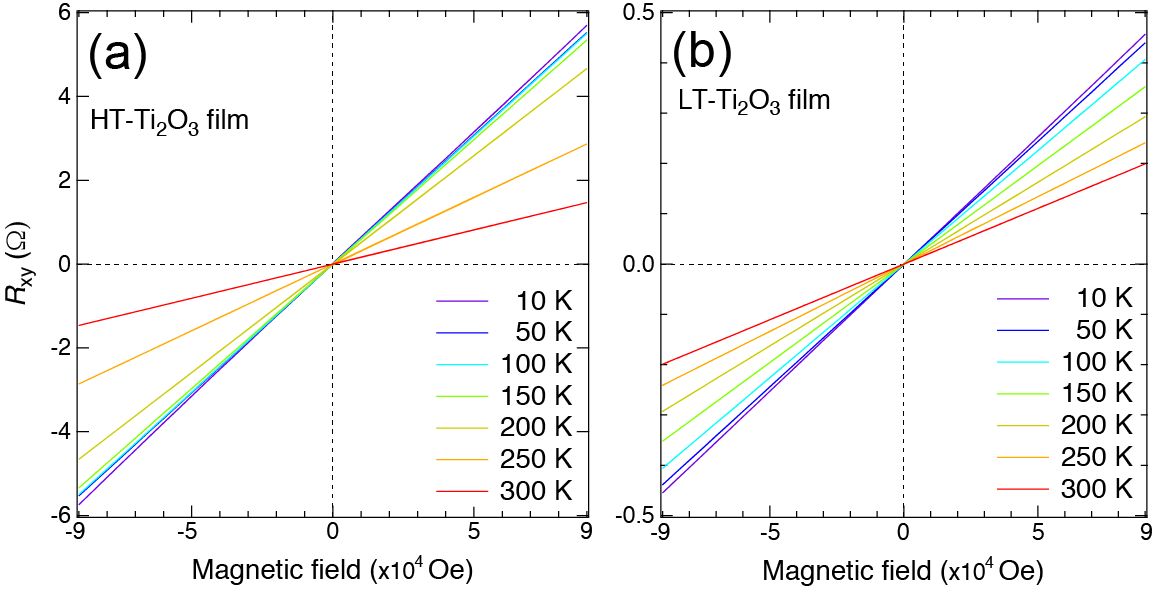


**Supplementary Figure 3. Hall resistance of the Ti_2_O_3_ films.** Magnetic-field dependence of the Hall resistance (*R*_xy_) for the (a) HT- and (b) LT-Ti_2_O_3_ films at various temperatures.

Electronic structures of Ti_2_O_3_ in DFT (+*U*) calculations

Supplementary Figure 4 shows the total density of states (DOS) near the Fermi level (*E*_F_) of bulk Ti_2_O_3_ from the DFT (+*U*) calculations. When Hubbard *U* was not introduced in the calculations, clear DOS was observed at *E*_F_, confirming the metallic states. The discrepancy between the DFT calculation and experiments was previously pointed out in the reference^[14]^. The electron correlation was crucial to reproduce the insulating electronic structures of bulk Ti_2_O_3_. The energy gap opened when the *U* value was more than 2.0 eV (Inset of Fig. 6). As shown in Supplementary Fig. 4, the energy gap of 80 meV appears at *E*_F_ when *U* = 2.2 eV. Apart from the energy gaps at *E*_F_, the shapes of DOS at the valence-band and conduction-band regions are similar for *U* = 0 and 2.2 eV.

**Supplementary Figure 4. Total DOS near *E*_F_ of bulk Ti_2_O_3_ in DFT +*U* calculations.** The *c*/*a* ratio is fixed 2.639 and *U* is set 0 eV (black) and 2.2 eV (red).

Relationship between the distance of Ti-Ti pair and MIT in DFT (+*U*) calculations

In bulk Ti_2_O_3_, the relationship between the distance of Ti-Ti pair and MIT was suggested from the XRD measurements including structural refinement [4, S4]. In order to ascertain the validity of our DFT (+*U*) calculations, we investigated the relationship between the ground states and the distance of Ti-Ti pair after the structural optimization. Supplementary Figure 5 shows the contour map of the distance of Ti-Ti pair as functions of *U* values and *c*/*a* ratios, together with the border of the MIT. The distance shortened when *U* increased and/or the *c*/*a* ratio decreased. The contour map revealed that the MIT occurred at the critical distance of ~2.53 Å, confirming that the distance of Ti-Ti pair was crucial for the electronic states of Ti_2_O_3_ in the DFT (+*U*) calculations. In experiments, the critical distance was estimated to be 2.645 Å^[4]^. Our calculation shows the shorter critical distance by 4.5 % than the experimental results.

**
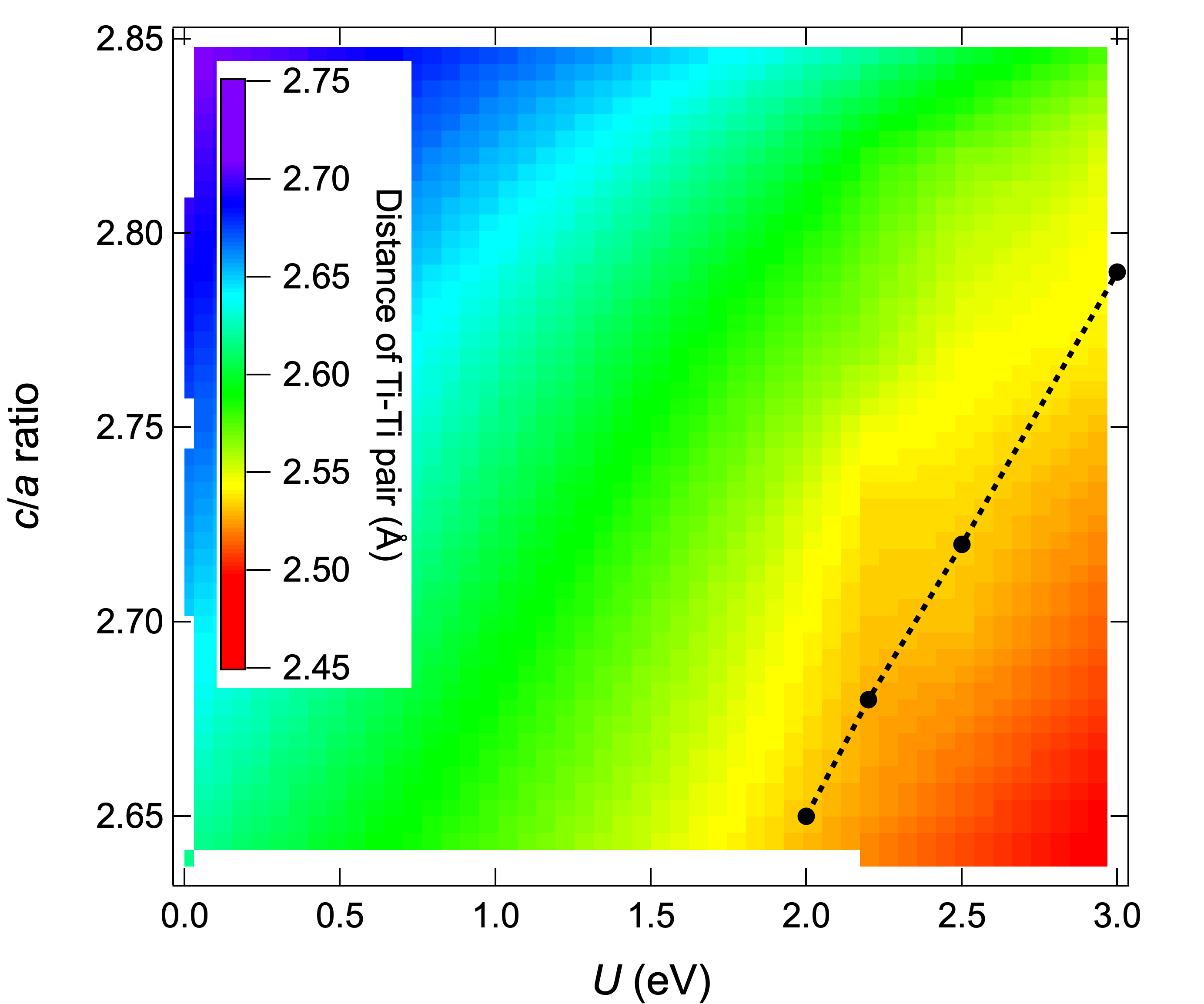
**

**Supplementary Figure 5. Contour map of the distance of Ti-Ti pair obtained from DFT + *U* calculations.** The dotted line indicates the border of the MIT on which the critical distance of Ti–Ti pair is ~2.53 Å.

Temperature-dependent XRD measurements to HT- and LT-Ti_2_O_3_ films

Temperature-dependent XRD measurements were performed with a four-circle diffractometer attached to a rotating anode X-ray generator. X-ray beam from a Mo target was monochromatized by a bent graphite monochromator. The sample temperature was controlled by a closed cycle He refrigerator. Supplementary Figure 6 shows the temperature dependence of the XRD contour maps for the HT- and LT-Ti_2_O_3_ films around α-Al_2_O_3_ 00012 reflections. We found that substrate and film reflections clearly shifted toward higher 2*θ* angles with decreasing temperature, indicating that the *c*-axis lattice constant became shorter at low temperatures. Supplementary Figure 7 shows the temperature dependence of the XRD contour maps for the HT- and LT-Ti_2_O_3_ films around α-Al_2_O_3_ 11-20 reflections. Here, the XRD measurements along [11-20] direction were carried out in the transverse geometry to obtain in-plane lattice information. In contrast to 00012 reflections, the peak shift of the α-Al_2_O_3_ 11-20 reflections was hardly detected owing to their broad peak width under the transverse geometry of the XRD measurements.

We further analyzed the XRD contour maps to obtain thermal expansion coefficients of the HT- and LT-Ti_2_O_3_ films. Supplementary Figure 8 shows the temperature dependence of the *a*- and *c*-axis lattice constants for the HT- and LT-Ti_2_O_3_ films. Here, we calibrated the peak positions of the films by referring to those of α-Al_2_O_3_ substrates under the assumption that α-Al_2_O_3_ substrates exhibit the thermal expansion coefficients identical to the previous report [S1]. In order to indicate the difference in the *a*- and *c*-axis lattice constants, the determined lattice constants are plotted in the same figure and on the same scale. As shown in Supplementary Fig. 8, the *c*-axis lattice constants of both films were systematically shortened with decreasing temperature and almost remained intact below 50 K. In contrast, the change of the *a*-axis lattice constants was relatively small around RT and would be within the experimental errors.

By using the lattice constants in the high-temperature regions (> 200 K), the thermal expansion coefficients along the *c*-axis (*α*_c_) and *a*-axis (*α*_a_) were estimated to be *α*_c_ = 1.86 ± 0.17 × 10^-5^ K^-1^ and *α*_a_ = 2.10 ± 2.97 × 10^-6^ K^-1^ for the HT-Ti_2_O_3_ film and to be *α*_c_ = 1.70 ± 0.11 × 10^-5^ K^-1^ and *α*_a_ = -0.37 ± 3.32 × 10^-6^ K^-1^ for the LT-Ti_2_O_3_ film. The *α*_a_ was smaller by an order of magnitude than the *α*_c_. In bulk Ti_2_O_3_, we estimated the thermal expansion coefficients to be *α*_c_ = 3.3 × 10^-5^ K^-1^ and *α*_a_ = -1.1 × 10^-6^ K^-1^ from the previous report (see Supplementary Figure 9) [4]. We found that the *α*_c_ of the films was approximately half of the *α*_c_ of the bulk specimen.


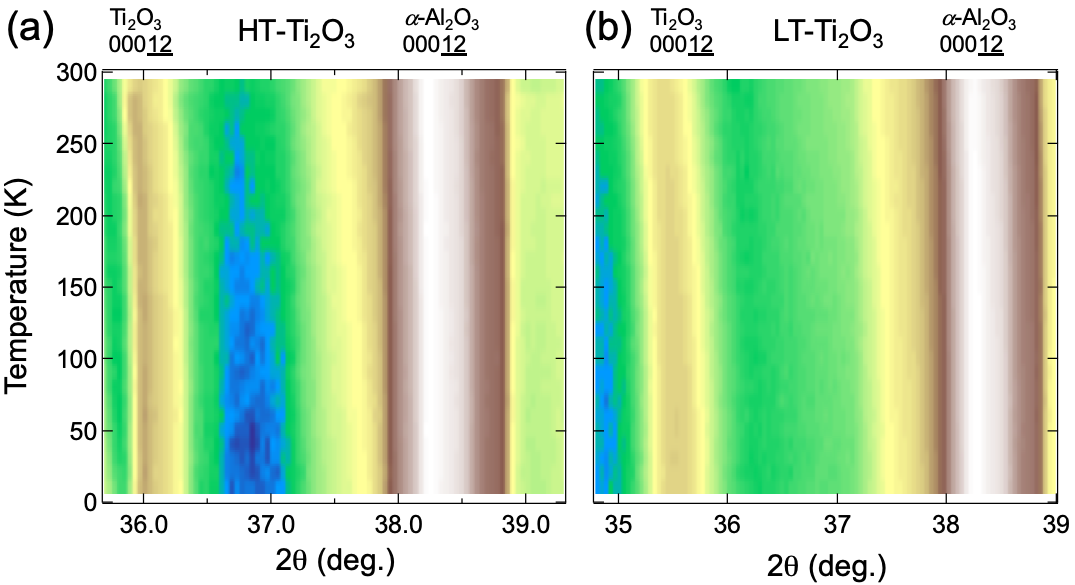


**Supplementary Figure 6. Temperature dependence of XRD contour maps.** The XRD contour maps for the (a) HT- and (b) LT-Ti_2_O_3_ films are taken around α-Al_2_O_3_ 00012 reflections.


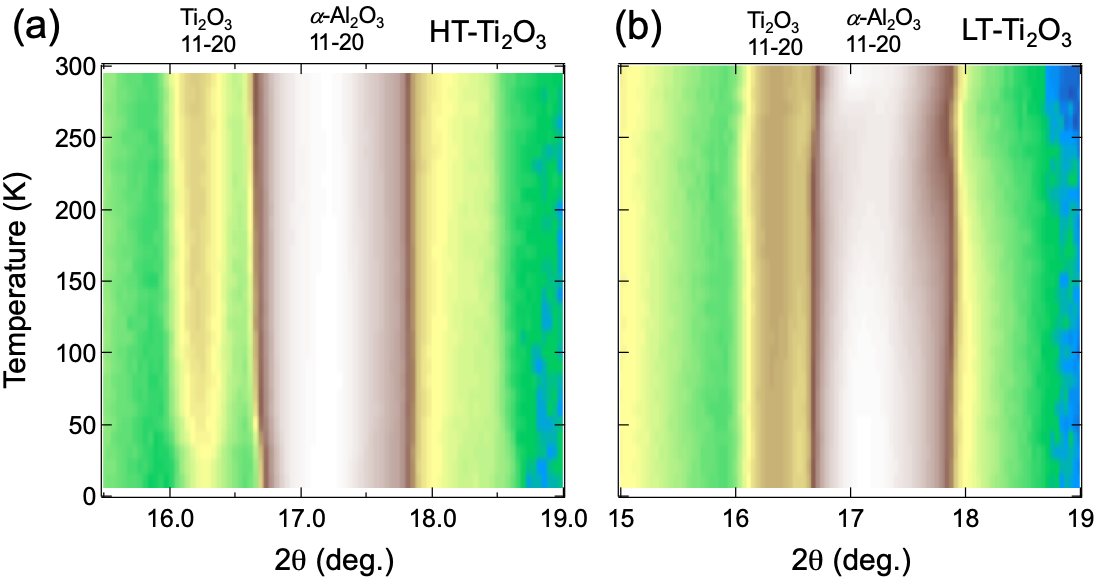


**Supplementary Figure 7. Temperature dependence of XRD contour maps.** The XRD contour maps for the (a) HT- and (b) LT-Ti_2_O_3_ films are taken around α-Al_2_O_3_ 11-20 reflections.


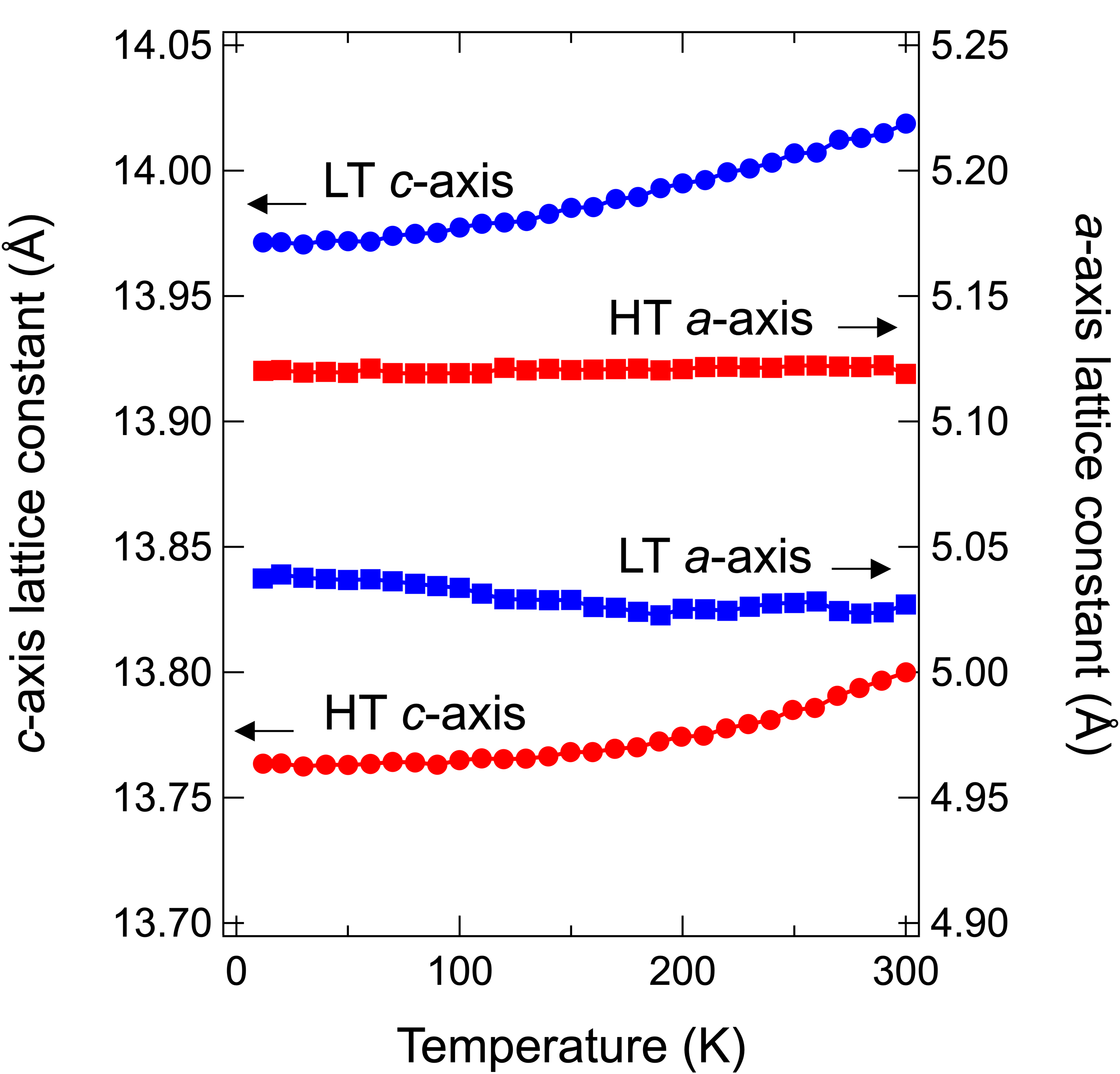


**Supplementary Figure 8. Temperature dependence of *a* and *c* lattice constants for the HT- and LT-Ti_2_O_3_ films.**

Thermal expansion coefficient in bulk Ti_2_O_3_

From the reported results for the bulk systems^[4]^, we estimated the thermal expansion coefficient (α) of Ti_2_O_3_ to discuss the lattice constants of the Ti_2_O_3_ films at low temperatures. Supplementary Figure 9 shows *a* and *c* lattice constants of bulk Ti_2_O_3_ from 300 to 800 K. The *a* (*c*) lattice constant monotonically decreased (increased) with the increase in temperature, resulting enhanced *c*/*a* ratios. Across the MIT in the temperature range of 400–600 K, the lattice constants were modulated significantly. From the temperature dependence of the lattice constants in metallic regions (above 600 K), we estimated α in bulk Ti_2_O_3_ in the following method.

In solids, temperature dependence of the lattice constants is simply described as follows:

*a*_T1_ = *a*_T0_×(1+αΔT), (Eq. S1)

where *a*_T_ is a lattice constant at temperature T, ΔT is the difference in the temperatures (= T_1_–T_0_), and α is the temperature independent thermal expansion coefficient. As Ti_2_O_3_ is an anisotropic material considering *a-* and *c-*axis directions, α also depends on the crystallographic axes. Therefore, we calculated α along both *a*- and *c*-axis directions (α_a_ and α_c_, respectively) using the data shown in Supplementary Fig. 9. From the least square fit of the temperature dependence of the lattice constants above 600 K, we obtained α_a_ = -1.08763 × 10^-6^ K^-1^ and α_c_ = 3.26323 × 10^-5^ K^-1^.

**Supplementary Figure 9. *a* and *c* lattice constants of bulk Ti_2_O_3_ from 300 to 800 K.** The data are taken from Ref. 4^[4]^.

Temperature dependence of the *c*/*a* ratios for Ti_2_O_3_ films estimated from the thermal expansion coefficients

Using the α obtained from the temperature-dependent XRD measurements (Supplementary Figs. 6–8) and the lattice constants at RT (Fig. 3), we estimated the *c*/*a* ratios for the HT- and LT-Ti_2_O_3_ films at low temperatures by the linear extrapolation of the values in high-temperature metallic region to low temperatures. We also estimated the *c*/*a* ratios of the films using the α obtained from bulk Ti_2_O_3_ (see Supplementary Fig. 9) as references. Supplementary Fig. 10 shows the results in comparison with the critical *c*/*a* ratio for the occurrence of MIT. Regardless of the choice of the thermal expansion coefficients, we obtained the qualitatively same conclusion. For the HT-Ti_2_O_3_ film, the *c*/*a* ratio reached the critical value of 2.68 at ~150 K using the α of the bulk reference and at ~ 50 K using the experimentally determined α. In contrast, the LT-Ti_2_O_3_ film did not reach the critical value even at the lowest temperature limit. Although these critical MIT temperatures are much lower than that revealed from the temperature dependence of resistivity (~250 K in Fig. 5a), the results demonstrate that the temperature dependent MIT in the HT-Ti_2_O_3_ films, as well as the difference in the ground states between the LT-Ti_2_O_3_ and HT- Ti_2_O_3_ films, is due to the temperature dependence of the crystal deformation.


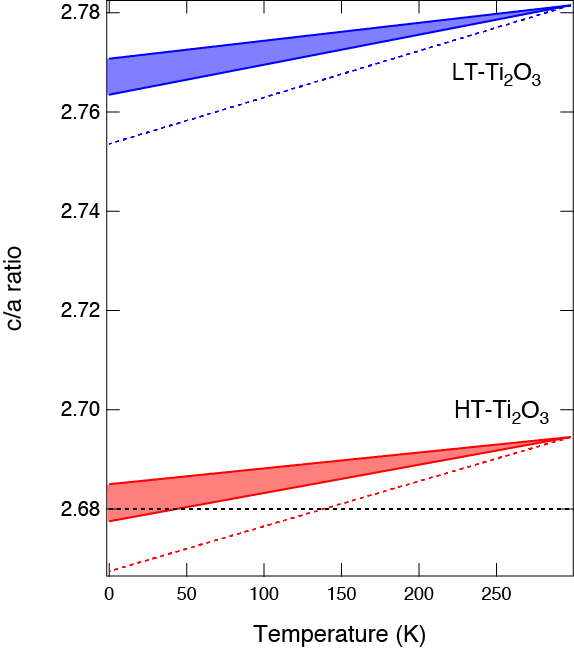


**Supplementary Figure 10. Temperature dependence of the *c*/*a* ratios for the HT- and LT-Ti_2_O_3_ films estimated from the thermal expansion coefficients obtained from experiments (filled regions) and from the bulk reference (dashed colored lines).** The dashed black line indicates the critical *c*/*a* ratios of 2.68 where the occurrence of MIT is expected from the DFT calculations.

Growth of TiO*_x_* films at different temperatures

We fabricated TiO*_x_* films at various substrate temperatures to obtain corundum-type Ti_2_O_3_ films with different domain sizes. However, another superconducting γ-Ti_3_O_5_ phase was more stable than the corundum-type Ti_2_O_3_ one in the middle temperatures. Supplementary Figure 11 shows out-of-plane XRD patterns of TiO*_x_* films grown at 500, 750, and 1000 °C. The films grown at 500 and 1000 °C were indicated as the LT- and HT-Ti_2_O_3_ films, respectively, in the main manuscript. The film grown at 750 °C showed superconducting γ-Ti_3_O_5_ phase confirmed by the XRD measurements, Raman spectroscopy, and temperature dependent resistivity measurements (Supplementary Figs. 11–14). Supplementary Figure 12 shows the Raman spectrum of the γ-Ti_3_O_5_ film measured using He-Ne laser. The γ-Ti_3_O_5_ film shows completely different spectral shape from those of the corundum-type Ti_2_O_3_ films. The Raman spectrum of the γ-Ti_3_O_5_ film was in good agreement with that of the Rh_2_O_3_-type Ti_2_O_3_ film reported by Li *et al*.^[24]^, suggesting that these superconducting films exhibit identical crystal structures. We note that the Raman spectrum of the γ-Ti_3_O_5_ film is different from that of γ-Ti_3_O_5_ bulk in the previous report^[S3]^. Moreover, the Raman spectrum we obtained from the γ-Ti_3_O_5_ film has not been reported in any bulk titanates. Formation of the γ-Ti_3_O_5_ phase was stable up to ~900 °C, which was also reported previously^[24, 40, S4]^.

Superconductivity in the γ-Ti_3_O_5_ film was confirmed from the temperature dependence of the resistivity at low temperatures (Supplementary Fig. 13). The superconducting transition temperature was 5.6 K under zero magnetic field, and the magnetic fields suppressed the superconducting phase.

When TiO_x_ films were grown at ~600 °C, the films were eutectic including Ti_2_O_3_ and γ-Ti_3_O_5_ phases, which was revealed by Raman spectroscopy. Supplementary Figure 14 shows microscopic images of the LT-Ti_2_O_3_, γ-Ti_3_O_5_, and eutectic films taken in the Raman apparatus. The different colors of the films suggest different titanate compositions. Red, blue, and mixture of blue and red spots were found on the surfaces of the LT-Ti_2_O_3_, γ-Ti_3_O_5_, and eutectic films, respectively. In addition, the Raman spectrum of the eutectic film suggested combination of those of γ-Ti_3_O_5_ and LT-Ti_2_O_3_, confirming their mixed states within the film.

According to aforementioned results, we concluded that the superconducting γ-Ti_3_O_5_ phase was stable in the middle growth temperatures. The corundum-type Ti_2_O_3_ films grown under 500 °C (LT-Ti_2_O_3_) and 1000 °C (HT-Ti_2_O_3_) were only discussed in the main manuscript.

**Supplementary Figure 12. Raman spectrum of the superconducting *γ*-Ti_3_O_5_ film.** The Raman spectrum of the superconducting Rh_2_O_3_-type Ti_2_O_3_ film is also shown for comparison^[24]^.


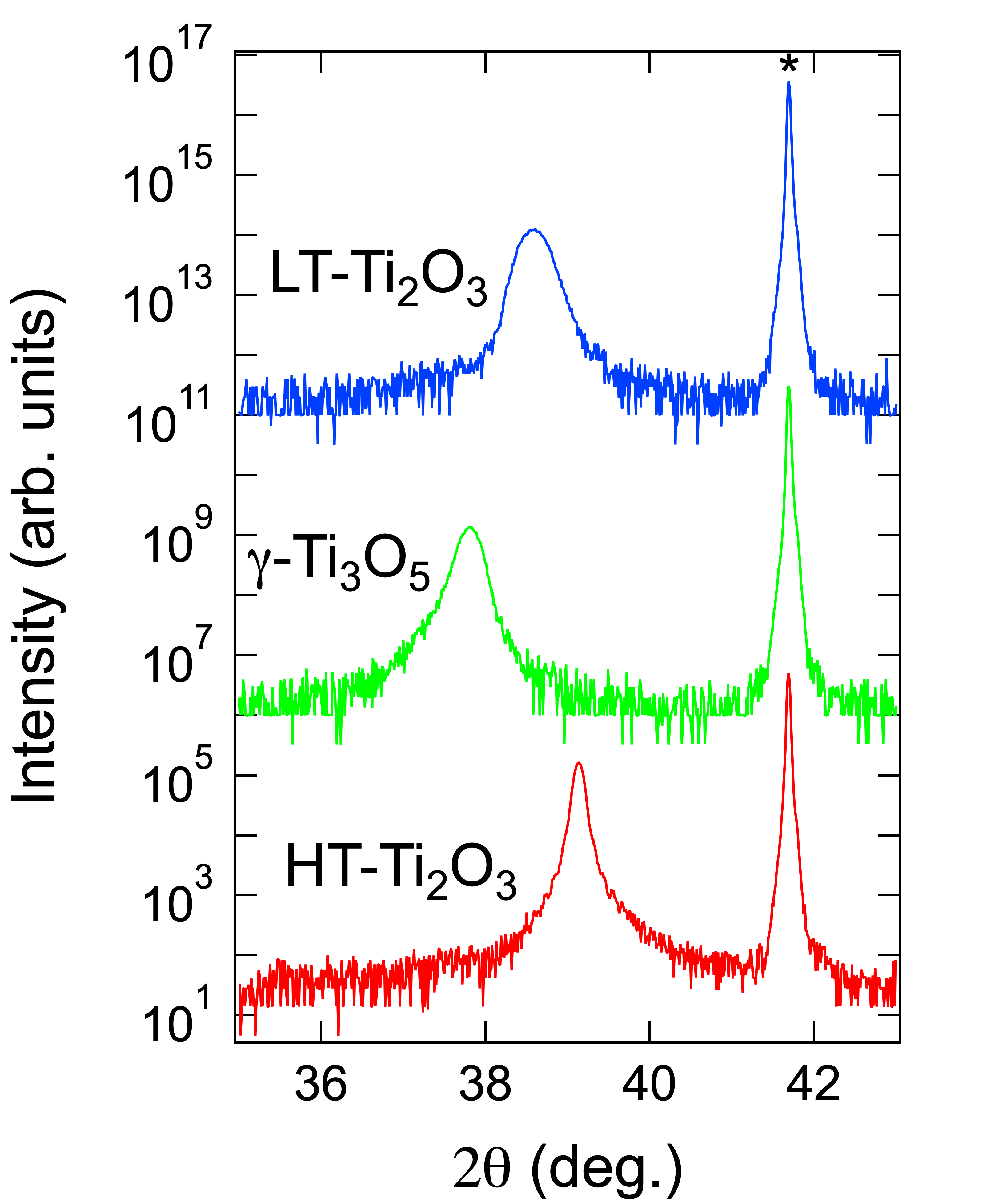


**Supplementary Figure 11. Out-of-plane XRD patterns.** The red, green, and blue colors indicate HT-Ti_2_O_3_, *γ*-Ti_3_O_5_, and LT-Ti_2_O_3_ films, respectively. The asterisk indicates *α*-Al_2_O_3_ 0006 reflections.


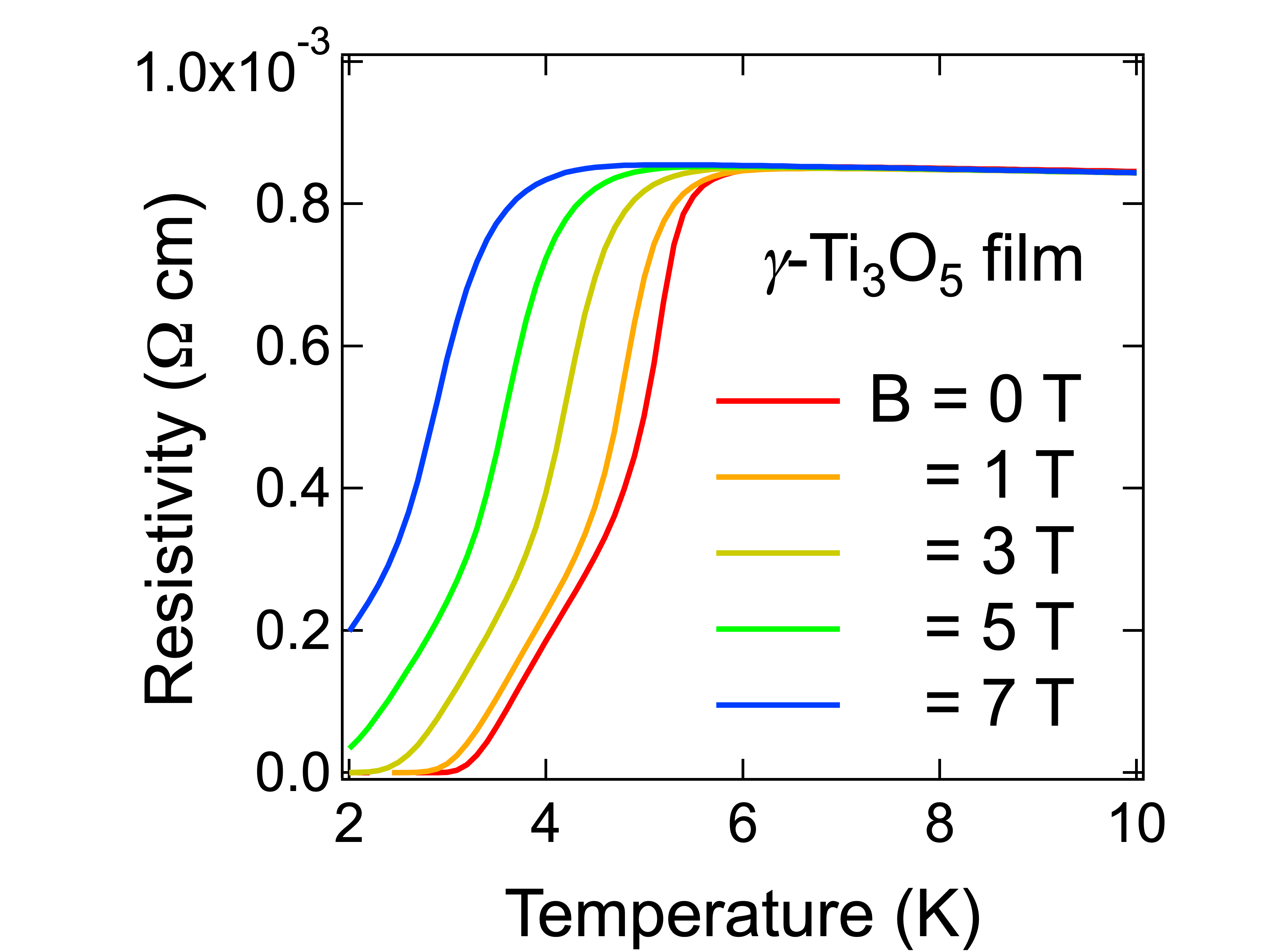


**Supplementary Figure 13. Temperature dependence of resistivity for the *γ*-Ti_3_O_5_ film at low temperatures under various magnetic fields.** The magnetic fields were applied perpendicular to the film surface.


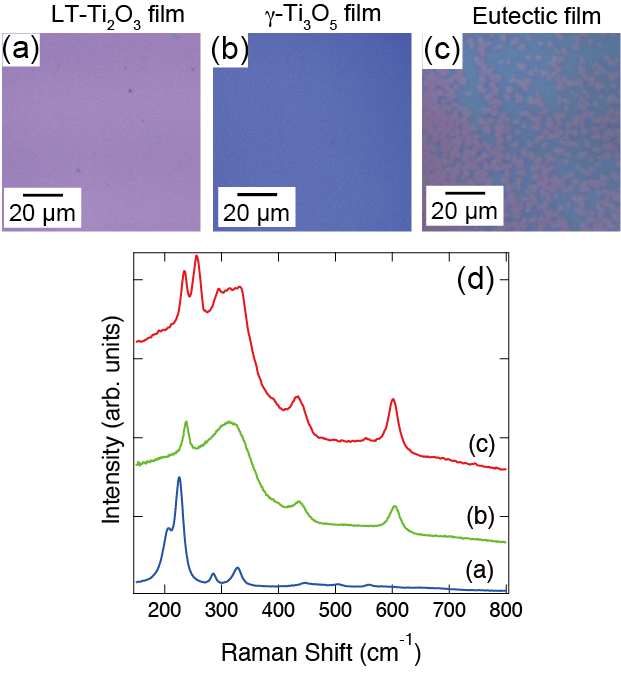


**Supplementary Figure 14. Microscopic images and Raman spectra of the titanate films.** Microscopic images of (a) LT-Ti_2_O_3_, (b) γ-Ti_3_O_5_, and (c) eutectic film surfaces. (d) Corresponding Raman spectra of the films.

**Supplementary Methods**

Computing details

Crystal structure of Ti_2_O_3_ is usually regarded as a corundum-type one as depicted in Supplementary Fig. 15(a). Lattice parameters of bulk Ti_2_O_3_ are *a* = 5.157 Å and *c* = 13.61 Å at RT, resulting in *c*/*a* = 2.639, which is much smaller than the isostructural *α*-Al_2_O_3_ (*a* = 4.759 Å, *c* = 12.99 Å, and *c*/*a* = 2.732 at RT)^[S2]^. The unit-cell volume of the trigonal Ti_2_O_3_ (*V*_t_) is as follows:

$V=\frac{\sqrt{3}}{2}a^{2}c$. (Eq. S2)

We calculated electronic structures of Ti_2_O_3_ assuming various *c*/*a* ratios (2.639 ≤ *c*/*a* ≤ 2.80), while keeping the unit-cell volume identical to that of the bulk (V_bulk_). The *c*/*a* ratio of 2.80 is large for bulk Ti_2_O_3_ but typical in V_2_O_3_^[15, 41]^. The *a* and *c* lattice constants in the case of *c*/*a* = *t* and *V*_t_ = V_bulk_ were calculated applying following equation:

$a=\sqrt[3]{\frac{{2V}_{bulk}}{\sqrt{3}t}}$, $c=\sqrt[3]{\frac{{2tV}_{bulk}}{\sqrt{3}}}$. (Eq. S3)

Since the trigonal unit cell contains several atoms (12 Ti and 18 O atoms), as shown in Supplementary Fig. 15(a), electronic structures were calculated in a rhombohedral primitive cell, as described in Supplementary Fig. 15(b), to reduce the computing cost. The lattice parameters of the rhombohedral unit cell were obtained from the trigonal parameters using the following equations:

$a_{r}=\frac{1}{3}\sqrt{\left( 3a^{2}+c^{2} \right)}$, (Eq. S4)

$\sin\frac{\alpha}{2}=\frac{3}{2}\left( 3+\frac{c^{2}}{a^{2}} \right)^{-\frac{1}{2}}$. (Eq. S5)

The rhombohedral lattice parameters of bulk Ti_2_O_3_ at RT are *a*_r_ = 5.426 Å, *α* = 56.741º.


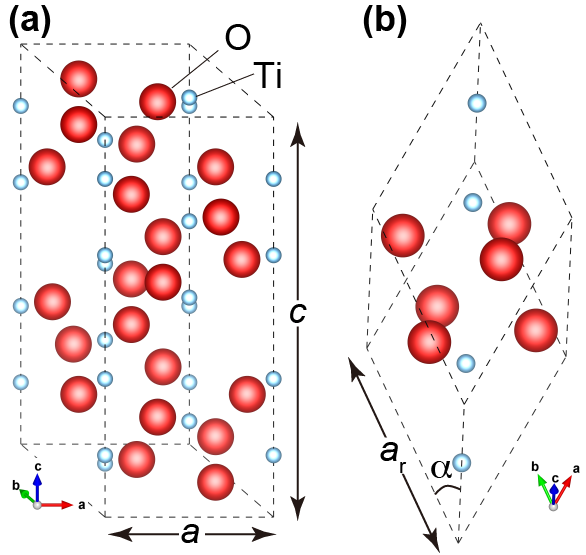


**Supplementary Figure 15. Schematic crystal structures of Ti_2_O_3_.** (a) Trigonal and (b) rhombohedral unit cells. The light blue and red spheres indicate Ti and O atoms, respectively. The trigonal lattice parameters *a* and *c*, and rhombohedral lattice parameters *a*_r_ and *α* are shown in (a) and (b), respectively. The schematics are drawn using VESTA^[S5]^.

**Supplementary Note 1**

Assignment of vibrational modes in Raman spectra

In Ti_2_O_3_, the *A*_1g_ and *E*_g_ vibrational modes cannot be assigned easily because the 1^st^ and 2^nd^ low-frequency modes are opposite to another corundum-type oxide of *α*-Al_2_O_3_^[28]^. In fact, their assignment was ambiguous in the previous reports^[11–13, 24, 25]^. In *α*-Al_2_O_3_, the 1^st^ and 2^nd^ low-frequency modes are assigned to the *E*_g_ and *A*_1g_ modes, respectively, determined from the polarization dependence of Raman spectra^[12, 13]^. Shin *et al*. studied polarization and photon-energy dependence of Raman spectra for Ti_2_O_3_. From the intensity modulations, the 1^st^ and 2^nd^ lowest-frequency modes were assigned to the *A*_1g_ and *E*_g_ modes, respectively, which was however opposite to those in *α*-Al_2_O_3_. From the resonant Raman effect, the 1^st^ and 2^nd^ low-frequency modes in our HT- and LT-Ti_2_O_3_ films were assigned to the *A*_1g_ and *E*_g_ modes, respectively, which was consistent with the previous report by Shin *et al.*^[12, 13]^.

**Supplementary Note 2**

Analysis of grain sizes in Ti_2_O_3_ films from the Scherrer equation

It is known that a mean grain size of a material can be estimated from XRD patterns using the Scherrer equation. The Scherrer equation is represented as follows:

*D* = *Kλ*/*β*cosθ, (Eq. S6)

where *D* is the mean size of the grains, *K* is a dimensionless shape factor whose value depends on the shape of grains, *λ* is the X-ray wavelength (λ = 1.5406 Å in this study), *β* is the FWHM of a peak in the 2*θ*–*θ* scans, and *θ* is the Bragg angle of a peak. Herein, we utilized *K* = 0.9 for simple estimation. We applied the Scherrer equation to out-of-plane XRD patterns of the HT- and LT-Ti_2_O_3_ films shown in Fig. 3a. The FWHMs of Ti_2_O_3_ 0006 reflection were 0.09° and 0.36° for the HT- and LT-Ti_2_O_3_ films after subtracting the instrumental 2*θ* broadening estimated from the substrate reflections. The obtained out-of-plane grain sizes were 94 and 23 nm for the HT- and LT-Ti_2_O_3_ films, respectively. The former was comparable to the film thickness and the latter was comparable to the in-plane grain sizes estimated from the AFM images (Fig. 4).

References

[S1] Lucht, M., Lerche, M., Wule, H. -C., Shvyd’ko, Y. V., Rüter, H. D., Gerdau, E. & Becker, P. Precise measurement of the lattice parameters of sapphire in the temperature range 4.5 K – 250 K using the Mössbauer wavelength standard. *J. Appl. Cryst.* **36**, 1075 (2003).

[S2] Rice, C. E. & Robinson, W. R. High-temperature crystal chemistry of Ti_2_O_3_: structural changes accompanying the semiconductor-metal transition. *Acta Cryst.* **B33**, 1342 (1977).

[S3] Li, Y., Bai, H., Zhai, J., Yi, W., Li, J., Yang, H., & Xi, G. Alternative to Noble Metal Substrates: Metallic and Plasmonic Ti_3_O_5_ Hierarchical Microspheres for Surface Enhanced Raman Spectroscopy. *Anal. Chem.* **91**, 4496 (2019).

[S4] Kurokwa, H., Yoshimatsu, K., Sakata, O. & Ohtomo, A. Effects of phase fraction on superconductivity of low-valence eutectic titanate films. *J. Appl. Phys.* **122**, 055302 (2017).

[S5] Momma, K. & Izumi, F. VESTA: a three-dimensional visualization system for electronic and structural analysis. *J. Appl. Cryst.* **41**, 653 (2008).
